# Clinical manifestations and risk factors of immune-related thyroid adverse events in patients treated with PD-1 inhibitors: a case-control study

**DOI:** 10.3389/fimmu.2025.1581057

**Published:** 2025-04-28

**Authors:** Pengfei Zhao, Jia Li, Lihong Yu, Wenming Ma, Ting Zhao

**Affiliations:** ^1^ Department of Clinical Pharmacy, Weifang People’s Hospital, Shandong Second Medical University, Weifang, Shandong, China; ^2^ Department of Adverse Drug Reaction Monitoring, Weifang Market Regulation Development Service Center, Weifang, Shandong, China

**Keywords:** hyperthyroidism, hypothyroidism, thyroiditis, PD-1 inhibitors, immune-related adverse events

## Abstract

**Background:**

Immune checkpoint inhibitors (ICPIs) have emerged as a powerful strategy to cancer treatment. However, while demonstrating antitumor efficacy, they can also induce a range of immune-related adverse events (irAEs). Immune-related thyroid dysfunction is one of the most common irAEs. This study aims to investigate the clinical characteristics and identify potential risk factors associated with PD-1 inhibitor-induced immune-related thyroid dysfunction in real-world.

**Methods:**

A retrospective analysis was conducted on the clinical data of cancer patients treated with PD-1 inhibitors at Weifang People’s Hospital from January 2021 to December 2024. The incidence, clinical subtypes, onset time, and prognostic outcomes of thyroid dysfunction were analyzed. Univariate and multivariate logistic regression analyses were performed to identify risk factors.

**Results:**

119 patients of PD-1 inhibitor-associated thyroid dysfunction were identified. The overall incidence of thyroid dysfunction was 2.97%, with hypothyroidism occurring in 1.20%, hyperthyroidism in 1.77%, and thyroiditis in 0.50% of patients. Tislelizumab exhibited the highest incidence at 3.48%, followed by camrelizumab at 3.10%, sintilimab at 2.24%, and toripalimab at 1.75%. The median time from the initiation of immunotherapy to the onset of thyroid dysfunction was 67 days, with hypothyroidism and hyperthyroidism developing at median times of 64.5 and 69 days, respectively. 77.31% of cases occurred within the first four months of immunotherapy. Female gender, lower baseline FT3 levels, history of targeted therapy, and baseline TgAb positivity were identified as independent risk factors for PD-1 inhibitor-associated thyroid dysfunction. Furthermore, higher baseline TSH levels, younger age, and treatment with tislelizumab or camrelizumab were associated with an increased risk of immune-related hypothyroidism, whereas lower baseline TSH levels were linked to a higher risk of immune-related hyperthyroidism.

**Conclusions:**

Close clinical and hormonal monitoring is recommended for patients with high-risk factors before and throughout the course of immunotherapy, particularly during the initial 2 to 4 months of PD-1 inhibitor treatment.

## 1 Introduction

Immune checkpoint inhibitors (ICPIs) represent a novel class of drugs employed in cancer immunotherapy. They function by activating T-cells, disrupting the immune tolerance to tumor antigens, thereby enabling T-cells to recognize and eliminate tumor cells. ICPIs significantly augment antitumor immune responses and improve the prognosis and survival outcomes for patients with malignant tumors (1). Currently, ICPIs have been approved for clinical application in a multitude of tumor types, encompassing non-small cell lung cancer, renal cell carcinoma, gastric cancer, colorectal cancer, hepatocellular carcinoma, and lymphomas. The most widely used ICPIs in clinical practice presently include three main categories: programmed death-1 (PD-1) inhibitors, cytotoxic T lymphocyte-associated antigen-4 (CTLA-4) inhibitors, and programmed death-ligand 1 (PD-L1) inhibitors.

Currently, ICPIs are widely used in the treatment of malignant tumors. However, with accumulating clinical data, the limitations of immunotherapy have become increasingly apparent. While ICPIs modulate the immune response to target and kill tumor cells, the overactivation of immune cells can also lead to autoimmune damage, resulting in immune-related adverse events (irAEs). IrAEs can affect multiple organ systems, with endocrine glands being particularly susceptible due to their rich blood supply and heightened sensitivity to immune responses. Endocrine-related irAEs mainly involve the thyroid, pituitary, pancreas, and adrenal glands, potentially causing various endocrine dysfunctions, such as thyroid dysfunction, hypophysitis, diabetes, and adrenal insufficiency. IrAEs can occur anytime during treatment and up to 3 months after treatment is completed (2).

**Figure 1:**
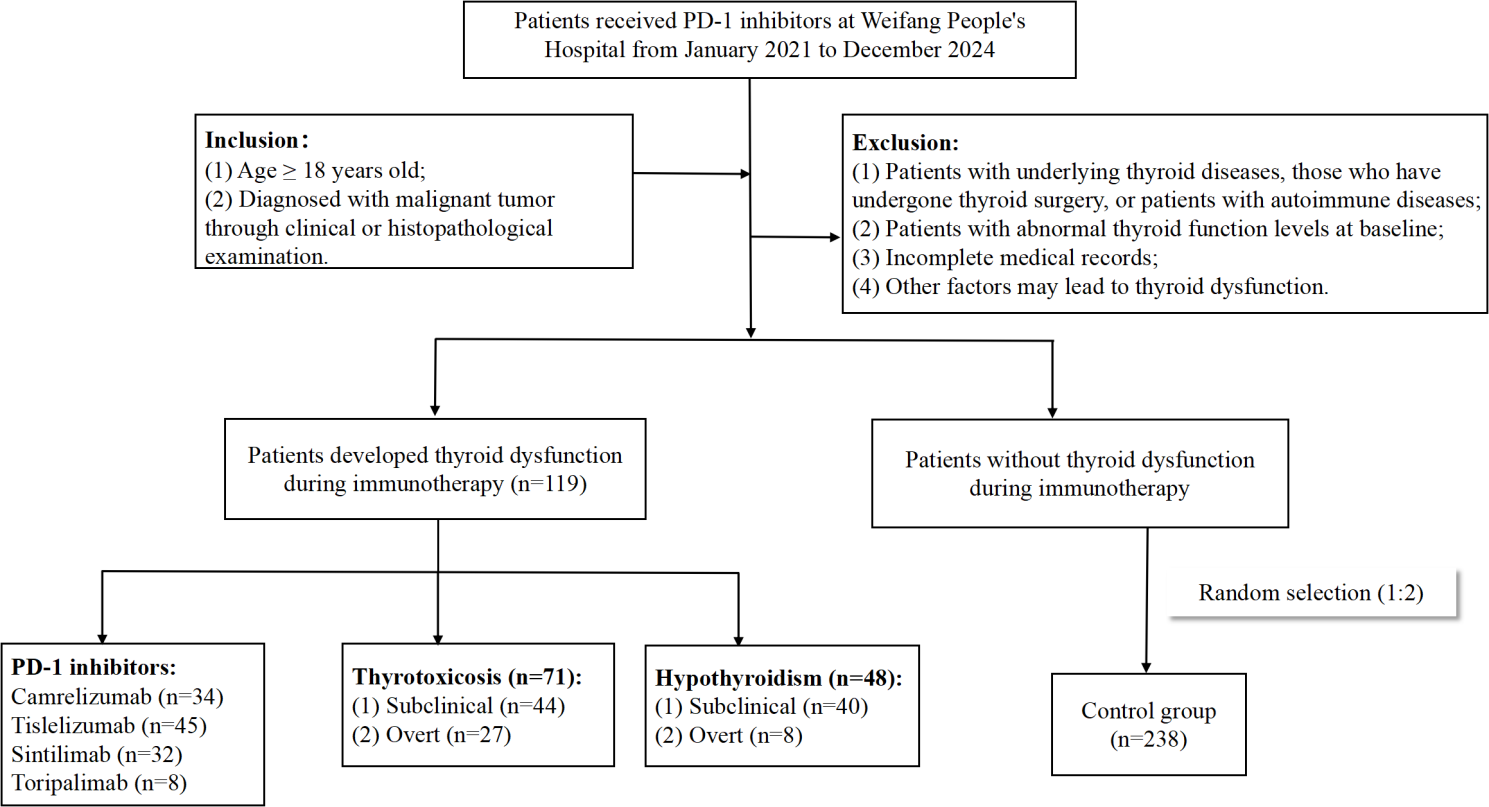
The technical flow chart of this study.

Thyroid injury is one of the most common endocrine adverse reactions associated with ICPIs, with an incidence ranging from 13.4% to 42.0% (3). Among these, thyroid dysfunction caused by PD-1 inhibitor is the most frequent, with an incidence of approximately 0% to 40%, which is higher than that observed with PD-L1 inhibitor (0% to 10%) and CTLA-4 inhibitor (0% to 7%) (4). Thyroid-related adverse events manifest in various forms, encompassing hypothyroidism, hyperthyroidism, thyroiditis, and even thyroid crisis, among which hypothyroidism exhibits the highest incidence. The clinical manifestations of thyroid dysfunction are often insidious in the early stage, with most patients remaining asymptomatic. Thyroid dysfunction is typically detected only during routine examinations, and it usually occurs weeks to months after starting immunotherapy. Patients with hypothyroidism often present with symptoms such as chills, fatigue, and general weakness, while those with severe disease may develop hypothyroid heart disease. Patients with hyperthyroidism typically present with hypermetabolic symptoms, such as heat intolerance, sweating, and weight loss. Severe hyperthyroidism can lead to thyroid crisis or thyrotoxic heart disease. Several studies suggested that immune-related thyroid dysfunction had a strong positive correlation with a favorable prognosis, yet they still affected the body’s metabolic, cardiovascular, and nervous systems. Severe manifestations include myxedema, heart and kidney failure, and potentially, even death (5, 6). Current literature on thyroid dysfunction caused by ICPIs remains limited, with most data derived from clinical studies. The involved subjects are rigorously screened, which may not fully reflect real-world scenarios. This study aims to investigate the clinical characteristics and associated factors of thyroid dysfunction in cancer patients treated with PD-1 inhibitors, so as to enhance clinicians’ understanding of immune-related thyroid dysfunction. The study process was outlined in **Figure 1**.

## 2 Methods

### 2.1 Study population

This single-center retrospective study was conducted on inpatients at Weifang People’s Hospital, a tertiary general hospital in China. Clinical records were acquired through the Hospital Information System spanning from January 1, 2021 to December 31, 2024. The Chinese Hospital Pharmacovigilance System (CHPS) was used to screen for patients with thyroid dysfunction based on predefined inclusion and exclusion criteria to generate early warning alerts. The monitoring depended on the availability of laboratory data in the patients’ medical records. Then, these alerts were subjected to manual review for validation. Patients with pathologically confirmed solid tumors, who were evaluated and agreed to receive PD-1 inhibitor therapy by professional oncologists, were enrolled in this study. Additionally, their levels of free triiodothyronine (FT3), free tetraiodothyronine (FT4), and thyroid stimulating hormone (TSH) were all within the normal range prior to PD-1 inhibitor treatment. We excluded patients with thyroid diseases, those who had undergone thyroid surgery, or patients with autoimmune diseases; patients with abnormal levels of FT3, FT4, or TSH prior to the initiation of immunotherapy; as well as patients with incomplete laboratory test results or medical records.

### 2.2 Data collection

For each enrolled patient, we collected the following data: age, gender, smoking and alcohol status, tumor type and stage, the dosage and frequency of PD-1 inhibitor administered, and the baseline clinical laboratory test results prior to PD-1 inhibitor initiation, including levels of FT3, FT4, and TSH, as well as the proportion of thyroglobulin antibody (TgAb) positivity and thyroid peroxidase antibody (TPOAb) positivity. The serum concentrations of thyroid hormones and antibodies were measured using chemiluminescent immunoassay with Siemens ADVIA Centaur^®^ XP system. Body mass index (BMI) can be determined through height and weight.

ICPIs-associated thyroid irAEs are subclassified based on the biochemical patterns of thyroid dysfunction. According to the 2016 guidelines issued by the American Thyroid Association (7), the four clinical types of thyroid dysfunction are defined as follows: ①Overt hyperthyroidism: TSH levels below the lower limit of the normal reference range, accompanied by FT3 or FT4 levels above the upper limit of the normal reference range; ②Subclinical hyperthyroidism: TSH levels below the lower limit of the normal reference range, with normal serum FT3 and FT4 concentrations; ③Overt hypothyroidism: TSH levels above the upper limit of the normal reference range, accompanied by FT3 or FT4 levels below the lower limit of the normal reference range; ④Subclinical hypothyroidism: TSH levels above the upper limit of the normal reference range, with normal serum FT3 and FT4 levels. For thyroid dysfunction group, we also evaluated the time from the initiation of PD-1 inhibitor therapy to the onset of thyroid dysfunction, encompassing both thyrotoxicosis and hypothyroidism, as well as the transition period from thyrotoxicosis to hypothyroidism. Additionally, we assessed the therapeutic interventions and subsequent outcomes.

### 2.3 Statistical analysis

Data was analyzed using SPSS Statistics Software version 26.0. Continuous variables with normal distribution were expressed as means ± standard deviation, while non-normally distributed variables were reported as medians with interquartile range (Q1, Q3). Normally distributed data was analyzed using the unpaired t-test for two independent groups and one-way analysis of variance (ANOVA) for multiple independent groups. For non-normally distributed data, the Mann-Whitney U test was employed to compare two independent groups, whereas the Kruskal-Wallis H test was used to evaluate differences across multiple independent groups. Categorical variables were presented in terms of counts and percentages. The duration from the initiation of PD-1 inhibitor treatment to the onset of thyroid dysfunction was assessed utilizing the Kaplan-Meier method. The χ² (Chi-square) test or the Fisher’s exact test was utilized to compare categorical variables between individuals with and without thyroid dysfunction. Variables found to be statistically significant in the univariate analysis were subjected to binary logistic regression analysis to identify independent risk factors. The adjusted odd ratios (OR) and 95% confidence intervals (CI) were computed for each variable. All statistical tests conducted were two-sided, and P-values less than 0.05 were deemed to indicate statistical significance.

## 3 Results

### 3.1 Basic patient information

**Figure 2:**
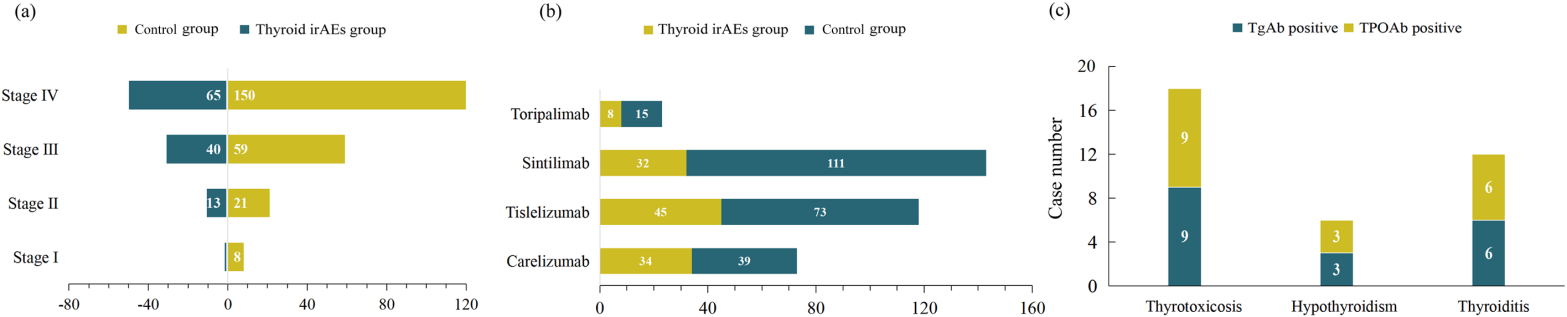
Number of patients with **(a)** different tumor stages, **(b)** different PD-1 inhibitors and **(c)** different types of thyroid dysfunction.

With the help of CHPS active surveillance system, we extracted data on 4012 patients who were hospitalized and received PD-1 inhibitor treatment at Weifang People’s Hospital from January 2021 to December 2024. Among them, 119 patients of PD-1 inhibitor-associated thyroid dysfunction were identified. The remaining patients were classified into the no-thyroid dysfunction group. Within the no-thyroid dysfunction group, a subset of 238 patients were randomly selected and designated as the control group. As can be seen from **Table 1**, among 357 patients analyzed, 270 patients were male and 87 patients were female, with a male to female ratio of 3.1: 1. The median age of the patients was 63.0 years. The primary tumor types of the included patients were diverse, mainly including lung cancer, gastric cancer and esophageal cancer. The tumor stages were predominantly stage III and stage IV (**Figure 2a**). Four PD-1 inhibitors were involved: 73 patients received camrelizumab, 118 patients received tislelizumab, 143 patients received sintilimab, and 23 patients received toripalimab (**Figure 2b**). As shown in **Table 2**, among the 119 patients, 71 cases had hyperthyroidism, including 27 cases of overt hyperthyroidism and 44 cases of subclinical hyperthyroidism; 48 cases had hypothyroidism, including 8 cases of overt hypothyroidism and 40 cases of subclinical hypothyroidism. In terms of prognosis, among patients with hyperthyroidism, 35 patients achieved normal thyroid function, and 1 patient was treated with anti-thyroid drug methimazole; among patients with hypothyroidism, 17 patients achieved normal thyroid function, and 12 patients were treated with levothyroxine sodium tablets (see **Table 3**).

**Table 1:** Comparison of demographic and clinical characteristics between thyroid dysfunction and control groups.

Characteristics	All patients (n=357)	Thyroid irAEs group (n=119)	Control Group (n=238)	Statistical value	P value
Gender, n (%)				9.848^a^	0.002
Male	270 (75.6%)	78 (65.5%)	192 (80.7%)		
Female	87 (24.4%)	41 (34.5%)	46 (19.3%)		
Age (years), M (Q25, Q75)	63.0 (56.5, 69.0)	62.0 (55.0, 69.0)	64.5 (57.0, 69.0)	12675.0b	0.106
BMI (kg/m2), M (Q25, Q75)	22.59 (20.22, 25.17)	22.79 (20.52, 25.40)	22.53 (20.04, 25.04)	10366.5^b^	0.489
Hypertension, n (%)	118 (33.0%)	32 (26.9%)	86 (36.1%)	3.063^a^	0.08
Diabetes mellitus, n (%)	45 (13.0%)	14 (11.8%)	31 (13.0%)	0.114^a^	0.735
Smoking, n (%)	201 (56.3%)	63 (52.9%)	138 (58.0%)	0.820^a^	0.365
Alcohol drinking, n (%)	168 (47.1%)	49 (41.2%)	119 (50.0%)	2.479^a^	0.115
Chemotherapy history, n (%)	341 (95.5%)	109 (91.6%)	232 (97.5%)	6.412^a^	0.011
Radiotherapy history, n (%)	97 (27.2%)	42 (35.3%)	55 (23.1%)	5.952^a^	0.015
Targeted therapy history, n (%)	98 (27.5%)	45 (37.8%)	53 (22.3%)	9.628^a^	0.002
Operation History, n (%)	194 (54.3%)	71 (59.7%)	123 (51.7%)	2.038^a^	0.153
PD-1 inhibitors, n (%)				14.73^a^	0.002
Carelizumab	73 (20.4%)	34 (28.6%)	39 (16.4%)		
Tislelizumab	118 (33.1%)	45 (37.8%)	73 (30.7%)		
Sintilimab	143 (40.1%)	32 (26.9%)	111 (46.6%)		
Toripalimab	23 (6.4%)	8 (6.7%)	15 (6.3%)		
Primary tumour type, n (%)				19.534^a^	0.002
Lung cancer	134 (37.5%)	47 (39.5%)	87 (36.6%)		
Gastric cancer	84 (23.5%)	16 (13.4%)	68 (28.6%)		
Esophagus cancer	59 (16.5%)	18 (15.1%)	41 (17.2%)		
Liver cancer	12 (3.4%)	7 (5.9%)	5 (2.1%)		
Colorectal cancer	10 (2.8%)	2 (1.7%)	8 (3.4%)		
Others	58 (16.2%)	29 (24.4%)	29 (12.2%)		
Baseline FT3 (pmol/L), mean ± SD	4.96 ± 0.81	4.77 ± 0.77	5.06 ± 0.82	-3.194c	0.002
Baseline FT4 (pmol/L), mean ± SD	16.43 ± 2.35	16.52 ± 2.47	16.39 ± 2.30	0.506^c^	0.613
Baseline TSH (μIU/mL), M (Q25, Q75)	1.60 (1.10, 2.30)	1.73 (1.10, 2.39)	1.58 (1.08, 2.22)	13298.5^b^	0.348
TgAb positive, n (%)	16 (5.5%)	12 (12.5%)	4 (2.1%)	13.517^a^	<0.001
TPOAb positive, n (%)	21 (7.19%)	12 (12.4%)	9 (4.6%)	5.838^a^	0.016

^a^Data was analyzed by *X* ^2^ test; ^b^Data was analyzed by Mann-Whitney U test; ^c^Data was analyzed by unpaired t-test.

BMI, body mass index; FT3, free triiodothyronine; FT4, free thyroxine; TSH, thyroid stimulating hormone; TPOAb, thyroid peroxidase antibody; TgAb, thyroglobulin antibody.

**Table 2:** Comparison of demographic and clinical characteristics between hyperthyroidism and hypothyroidism groups.

Characteristics	Hyperthyroidism (n=71)		Hypothyroidism (n=48)		Statistical value	P value
	Subclinical (n=44)	Overt (n=27)	Subclinical (n=40)	Overt (n=8)		
Gender, n (%)					0.941^a^	0.815
Male	29 (65.9%)	18 (66.7%)	27 (67.5%)	4 (50.0%)		
Female	15 (34.1%)	9 (33.3%)	13 (32.5%)	4 (50.0%)		
Age (years), mean ± SD	63.48 ± 9.90	60.85 ± 8.67	60.23 ± 8.66	55.13 ± 14.95	1.331b	0.284
BMI (kg/m2), M (Q25, Q75)	23.01 (20.20, 25.36)	23.51 (21.39, 26.97)	22.53 (21.64, 24.79)	20.34 (17.38, 23.39)	4.598c	0.204
PD-1 inhibitors, n (%)					13.943d	0.092
Carelizumab	7 (15.9%)	13 (48.1%)	13 (32.5%)	1 (12.5%)		
Tislelizumab	15 (34.1%)	9 (33.3%)	16 (40.0%)	5 (62.5%)		
Sintilimab	18 (40.9%)	4 (14.8%)	8 (20.0%)	2 (25.0%)		
Toripalimab	4 (9.1%)	1 (3.7%)	3 (7.5%)	0		
Primary tumor types, n (%)					24.115^d^	0.063
Lung cancer	23 (52.3%)	11 (40.7%)	10 (25.0%)	3 (37.5%)		
Gastric cancer	8 (18.2%)	2 (7.4%)	4 (10.0%)	2 (25.0%)		
Esophagus cancer	1 (2.3%)	5 (18.5%)	11 (27.5%)	1 (12.5%)		
Liver cancer	0	1 (3.7%)	5 (12.5%)	1 (12.5%)		
Colorectal cancer	1 (2.3%)	1 (3.7%)	0	0		
Others	11 (25.0%)	7 (25.9%)	10 (25.0%)	1 (12.5%)		
Tumor stage, n (%)					9.668^d^	0.366
①	1 (2.3%)	0	0	0		
②	4 (9.1%)	3 (11.1%)	5 (12.5%)	1 (12.5%)		
③	10 (22.7%)	13 (48.1%)	13 (32.5%)	4 (50.0%)		
④	29 (65.9%)	11 (40.7%)	22 (55.0%)	3 (37.5%)		
Baseline FT3 (pmol/L), mean ± SD	4.78 ± 0.85	4.92 ± 0.73	4.61 ± 0.76	5.00 ± 0.38	1.146^b^	0.334
Baseline FT4 (pmol/L), mean ± SD	17.00 ± 2.35	16.52 ± 2.60	16.03 ± 2.37	16.37 ± 3.16	1.091^b^	0.356
Baseline TSH (μIU/mL), M (Q25, Q75)	1.20 (0.76, 1.67)	1.76 (1.10, 2.39)	2.24 (1.50, 3.30)	1.94 (1.53, 2.75)	28.784^c^	<0.001
TgAb positive, n (%)	1 (3.0%)	8 (36.4%)	3 (8.8%)	0	12.029^d^	0.003
TPOAb positive, n (%)	3 (9.1%)	6 (27.3%)	2 (5.9%)	1 (12.5%)	5.467^d^	0.121
Progression to hypothyroidism, n (%)	3 (6.8%)	17 (63.0%)	Not applicable	Not applicable	Not applicable	Not applicable

^a^Data was analyzed by *X* ^2^ test; ^b^Data was analyzed by one-way ANOVA statistical analysis; ^c^Data was analyzed by Kruskal-Wallis H test; ^d^Data was analyzed by Fisher’s exact test.

BMI, body mass index; FT3, free triiodothyronine; FT4, free thyroxine; TSH, thyroid stimulating hormone; TPOAb, thyroid peroxidase antibody; TgAb, thyroglobulin antibody.

**Table 3:** Comparison of demographic and clinical characteristics between hyperthyroidism or hypothyroidism and control groups.

Characteristics	Control Group (n=238)	Thyroid irAEs group (119)					
		Hyperthyroidism (n=71)	Statistical value	P value	Hypothyroidism (n=48)	Statistical value	P value
Gender, n (%)			6.539^a^	0.011		6.02^a^	0.014
Male	192 (80.7%)	47 (66.2%)			31 (64.6%)		
Female	46 (19.3%)	24 (33.8%)			17 (35.4%)		
Age (years), M (Q25, Q75)	64.5 (57.0, 69.0)	62.0 (56.0,70.0)	8177.5^b^	0.681	59.0 (53.0, 66.8)	4497.5^b^	0.02
BMI (kg/m2), M (Q25, Q75)	22.53 (20.04, 25.04)	23.24 (20.28, 25.61)	6389.5^b^	0.285	22.2 (20.6, 24.3)	3844.0^b^	0.874
Hypertension, n (%)	86 (36.1%)	20 (28.2%)	1.540^a^	0.215	12 (25.0%)	2.199^a^	0.138
Diabetes mellitus, n (%)	31 (13.0%)	8 (11.3%)	0.153^a^	0.696	6 (12.5%)	0.01^a^	0.921
Smoking, n (%)	138 (58.0%)	39 (54.9%)	0.208^a^	0.648	24 (50.0%)	1.037^a^	0.309
Alcohol drinking, n (%)	119 (50.0%)	29 (40.8%)	1.837^a^	0.175	20 (41.7%)	1.110^a^	0.292
PD-1 inhibitors, n (%)			7.306^a^	0.063		11.886^a^	0.008
Carelizumab	39 (16.4%)	20 (28.2%)			14 (29.2%)		
Tislelizumab	73 (30.7%)	24 (33.8%)			21 (43.8%)		
Sintilimab	111 (46.6%)	22 (31.0%)			10 (20.8%)		
Toripalimab	15 (6.3%)	5 (7.0%)			3 (6.3%)		
Primary tumour types, n (%)			15.239d	0.007		22.448^a^	<0.001
Lung cancer	87 (36.6%)	34 (47.9%)			13 (27.1%)		
Gastric cancer	68 (28.6%)	10 (14.1%)			6 (12.5%)		
Esophagus cancer	41 (17.2%)	6 (8.5%)			12 (25.0%)		
Liver cancer	5 (2.1%)	1 (1.4%)			6 (12.5%)		
Colorectal cancer	8 (3.4%)	2 (2.8%)			0 (0.00%)		
Others	29 (12.2%)	18 (25.4%)			11 (22.9%)		
Tumor stage, n (%)			2.370^d^	0.499		4.247^d^	0.211
①	8 (3.4%)	1 (1.4%)			0 (0.00%)		
②	21 (8.8%)	7 (9.9%)			6 (12.5%)		
③	59 (24.8%)	23 (32.4%)			17 (35.4%)		
④	150 (63.0%)	40 (56.3%)			25 (52.1%)		
Baseline FT3 (pmol/L), mean ± SD	5.06 ± 0.82	4.83 ± 0.80	-2.041c	0.042	4.68 ± 0.73	-3.003^c^	0.003
Baseline FT4 (pmol/L), mean ± SD	16.39 ± 2.30	16.82 ± 2.44	1.364^c^	0.173	16.08 ± 2.48	-0.825^c^	0.41
Baseline TSH (μIU/mL), M (Q25, Q75)	1.58 (1.08, 2.22)	1.40 (0.85, 1.92)	7148.0^b^	0.049	2.12 (1.50, 3.06)	3548.5^b^	<0.001
TgAb positive, n (%)	4 (2.1%)	9 (16.4%)	15.041^d^	<0.001	3 (7.3%)	2.565^d^	0.103
TPOAb positive, n (%)	9 (4.6%)	9 (16.4%)	7.191d	0.006	3 (7.1%)	0.421^d^	0.45
Clinical prognosis, n (%)							
Recovery, n (%)	Not applicable	35 (49.3%)	Not applicable	Not applicable	17 (35.4%)	Not applicable	Not applicable
Taking medication, n (%)	Not applicable	1 (1.41%)	Not applicable	Not applicable	12 (25.0%)	Not applicable	Not applicable

^a^Data was analyzed by *X* ^2^ test; ^b^Data was analyzed by Mann-Whitney U test; ^c^Data was analyzed by unpaired t-test; ^d^Data was analyzed by Fisher’s exact test.

BMI, body mass index; FT3, free triiodothyronine; FT4, free thyroxine; TSH, thyroid stimulating hormone; TPOAb, thyroid peroxidase antibody; TgAb, thyroglobulin antibody.

### 3.2 Onset time and incidence of thyroid dysfunction

**Figure 3:**
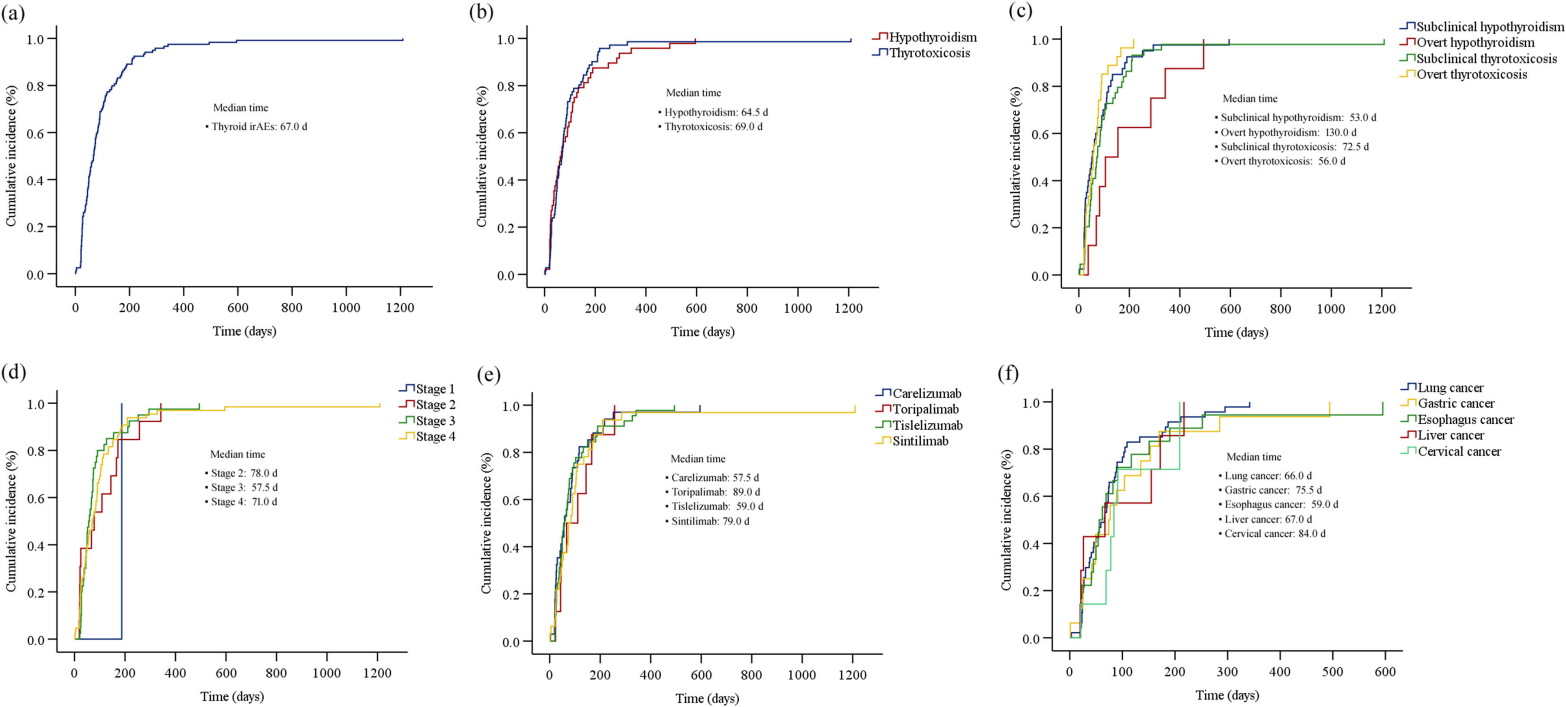
The cumulative incidence curve of the time to onset of thyroid dysfunction (biochemical detection) after initiation of PD-1 inhibitor therapy: **(a)** overall thyroid dysfunction; **(b)** hypothyroidism and hyperthyroidism; **(c)** four subtypes of thyroid dysfunction; **(d)** different tumor stages; **(e)** different PD-1 inhibitors; **(f)** different primary tumor types.

**Figure 4:**
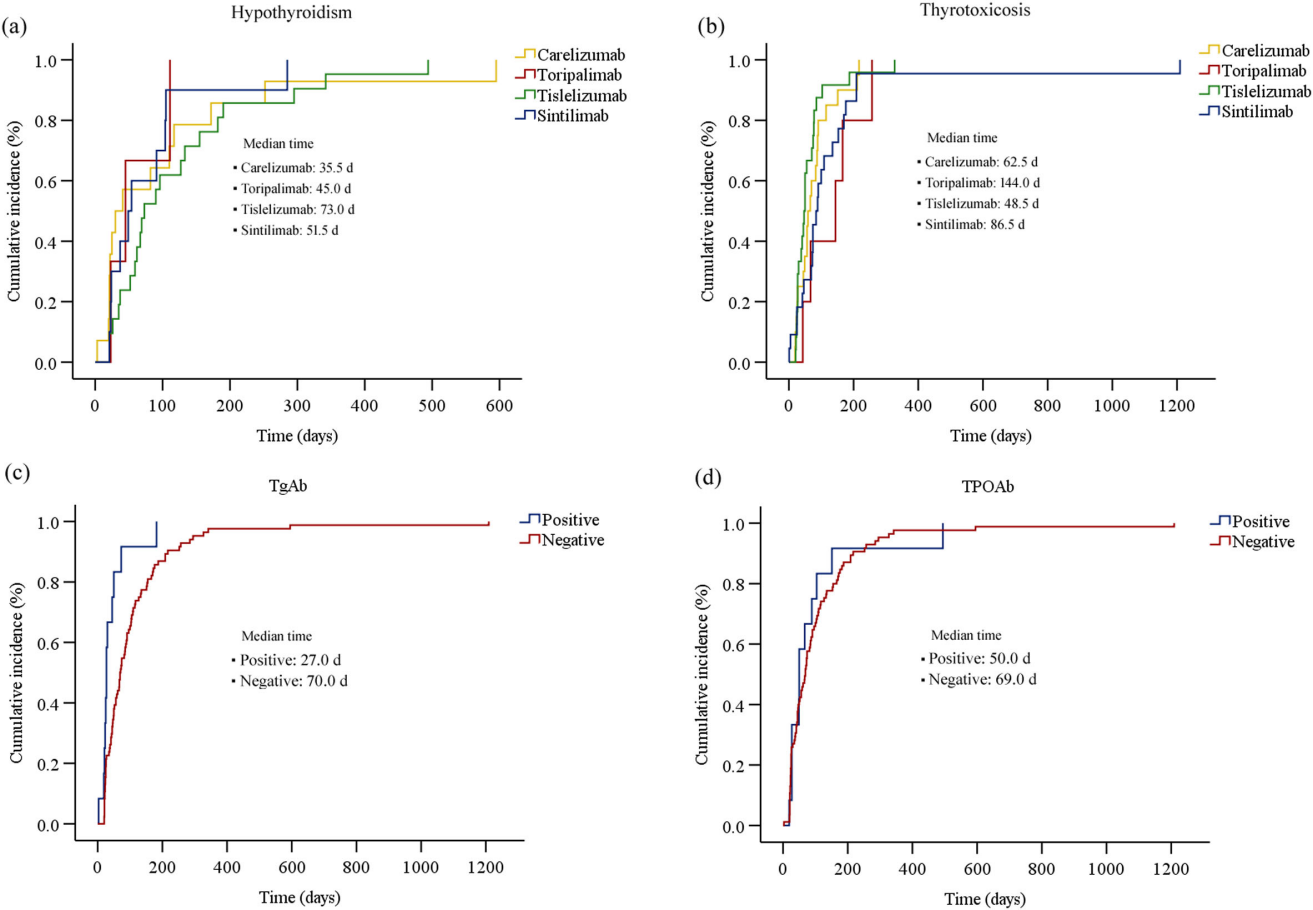
The cumulative incidence curve of the time to onset of thyroid dysfunction (biochemical detection) after initiation of PD-1 inhibitor therapy: **(a)** hypothyroidism; **(b)** thyrotoxicosis; **(c)** thyroid dysfunction for patients with different TgAb levels; **(d)** thyroid dysfunction for patients with different TPOAb levels.

**Figure 5:**
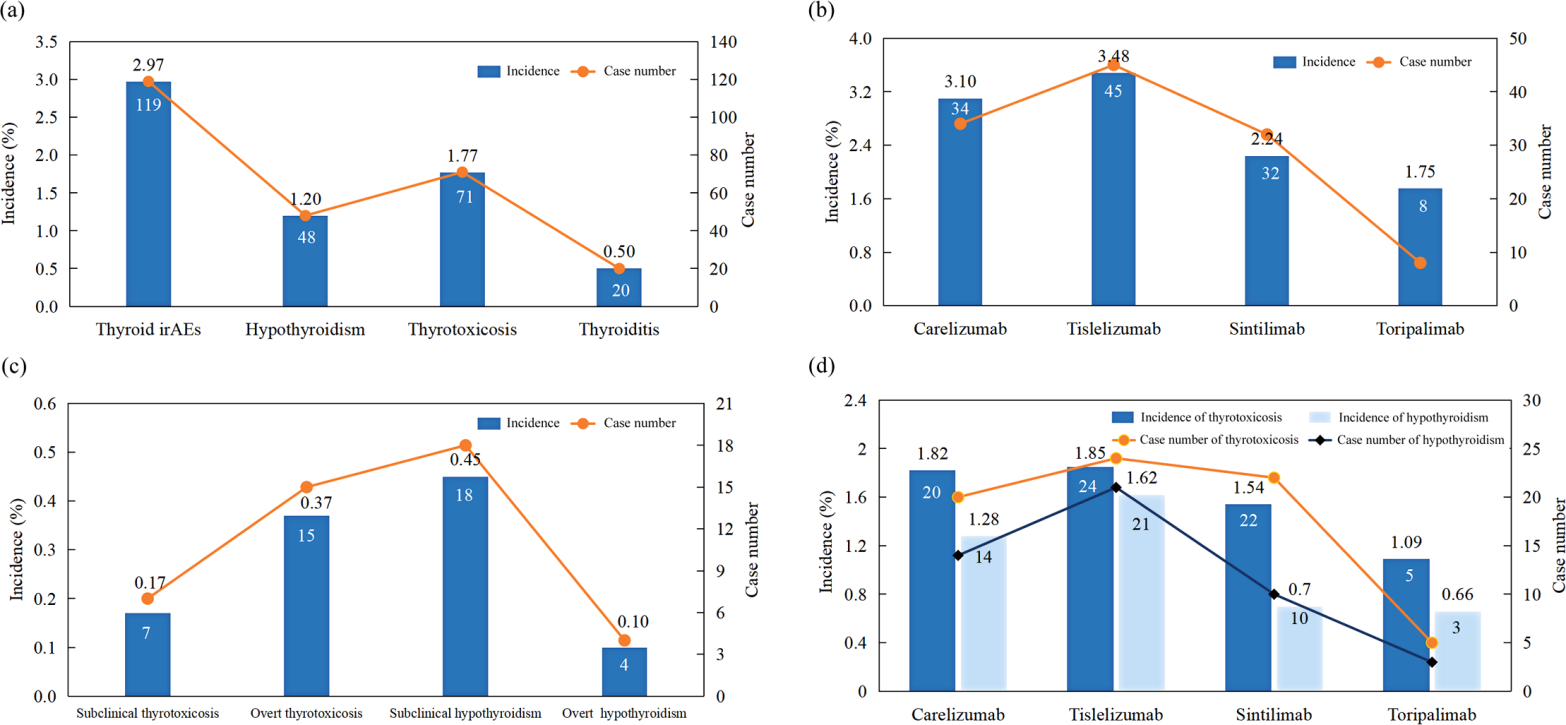
Incidence of **(a)** different types of thyroid dysfunction; **(b)** thyroid dysfunction induced by different PD-1 inhibitors; **(c)** different subtypes of thyroid dysfunction; **(d)** hypothyroidism and thyrotoxicosis in patients treated with different PD-1 inhibitors.

The Kaplan-Meier curves illustrating the time to the onset of thyroid dysfunction following the initiation of PD-1 inhibitor therapy were presented in **Figures 3** and **4**. Overall, the median time from the commencement of immunotherapy to the manifestation of thyroid dysfunction was 67 days. The median times to the onset of hypothyroidism and hyperthyroidism were 64.5 days and 69 days, respectively. For different PD-1 inhibitors, toripalimab was found to have the longest median time to onset of thyroid dysfunction at 89 days, followed by sintilimab at 79 days, tislelizumab at 59 days, and camrelizumab at 57.5 days (**Figure 3**). The median times to the onset of hypothyroidism and hyperthyroidism were 35.5 days and 62.5 days for camrelizumab, 45 days and 144 days for toripalimab, 73 days and 48.5 days for tislelizumab, and 51.5 days and 86.5 days for sintilimab, respectively. The levels of TgAb and TPOAb also significantly influenced the onset time of thyroid dysfunction. The median time of thyroid dysfunction was 27 days in TgAb positive patients and 70 days in TgAb negative patients. For TPOAb, the median onset time was 50 days in patients with positive TPOAb and 69 days in patients with negative TPOAb (**Figure 4**). Overall, in this study, the incidence of PD-1 inhibitor-related thyroid dysfunction was 2.97% (119/4012×100%), with hypothyroidism at 1.20%, hyperthyroidism at 1.77%, and thyroiditis at 0.50%. Among different PD-1 inhibitors, tislelizumab had the highest incidence of thyroid injury at 3.48%, followed by camrelizumab at 3.10%, sintilimab at 2.24%, and toripalimab at 1.75%. The incidence of hypothyroidism and hyperthyroidism were respectively 1.28% and 1.82% for camrelizumab, 1.62% and 1.85% for tislelizumab, 0.70% and 1.54% for sintilimab, 0.66% and 1.09% for toripalimab (**Figure 5**).

### 3.3 Characteristics of patients with immune-related thyroiditis

**Figure 6:**
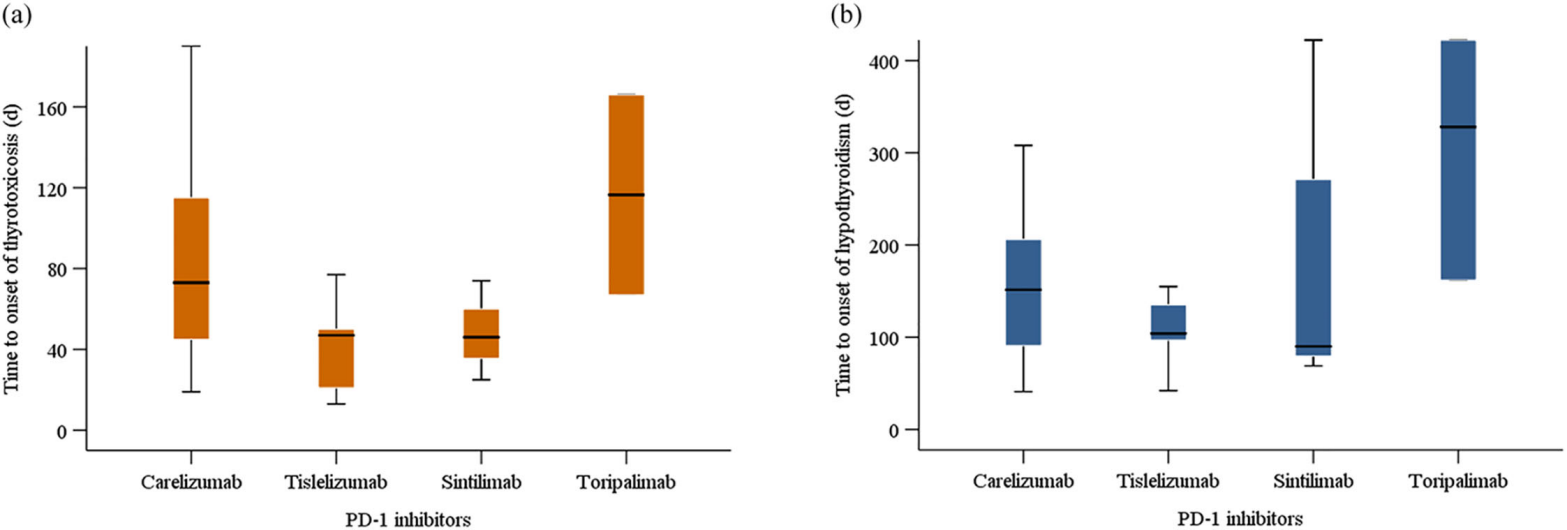
Time to onset of **(a)** thyrotoxicosis and **(b)** hypothyroidism for patients with thyroiditis.

Among the 71 patients initially diagnosed with hyperthyroidism, 20 gradually developed hypothyroidism, with a disease progression resembling thyroiditis accompanied by transient thyrotoxicosis. **Table 4** described the baseline characteristics of these 20 patients. There were 15 males and 5 females, yielding a male-to-female ratio of 3:1. The median age of the patients was 61 years. 10 patients received camrelizumab treatment, 5 patients received tislelizumab, 3 patients received sintilimab, and 2 patients received toripalimab. The median baseline TSH was 1.6 μIU/mL. The median TSH at the onset of thyrotoxicosis and hypothyroidism were 0.03 μIU/mL and 10.2 μIU/mL, respectively. The median time to onset of thyrotoxicosis and hypothyroidism were 57.5 days and 139.0 days, respectively. The median duration of the thyrotoxicosis phase was 68.0 days. For different PD-1 inhibitors, the median time to the onset of hyperthyroidism and hypothyroidism were respectively 73 days and 151.5 days for camrelizumab, 47 days and 104 days for tislelizumab, 46 days and 90 days for sintilimab, 116.5 days and 328 days for toripalimab (**Figure 6**). Of the 20 patients, 12 patients initiated levothyroxine sodium tablet treatment upon developing hypothyroidism. By the end of the follow-up period, 5 patients had recovered normal thyroid function. One patient started methimazole treatment after the onset of thyrotoxicosis, developed hypothyroidism 65 days later, and then discontinued methimazole and initiated levothyroxine sodium tablet treatment until the thyroid function returned to normal.

**Table 4:** Demographic and clinical characteristics of patients with thyroiditis.

Characteristics	
Gender, n (%)	
Male	15 (75.0%)
Female	5 (25.0%)
Age (years), median (IQR)	61.0 (56.0-68.0)
BMI (kg/m2), median (IQR)	23.8 (22.8-27.7)
Baseline FT3 (pmol/L), median (IQR)	4.8 (4.2-5.3)
Baseline FT4 (pmol/L), median (IQR)	16.2 (14.1-18.7)
PD-1 inhibitors, n (%)	
Carelizumab	10 (50.0%)
Tislelizumab	5 (25.0%)
Sintilimab	3 (15.0%)
Toripalimab	2 (10.0%)
Primary tumour types, n (%)	
Lung cancer	6 (30.0%)
Gastric cancer	3 (15.0%)
Esophagus cancer	4 (20.0%)
Tumor stage, n (%)	
II	2 (10.0%)
III	9 (45.0%)
IV	9 (45.0%)
TgAb positive, n (%)	6 (30.0%)
TPOAb positive, n (%)	6 (30.0%)
Baseline TSH (μIU/mL), median (IQR)	1.6 (1.1-2.2)
TSH at thyrotoxicosis (μIU/mL), median (IQR)	0.03 (0.01-0.10)
TSH at hypothyroidism (μIU/mL), median (IQR)	10.2 (5.6-40.8)
Time to onset of thyrotoxicosis (d), median (IQR)	57.5 (30.0-88.5)
Time to onset of hypothyroidism (d), median (IQR)	139.0 (90.3-202.8)
Time from thyrotoxicosis to hypothyroidism (d), median (IQR)	68.0 (43.0-102.5)

BMI, body mass index; FT3, free triiodothyronine; FT4, free thyroxine; TSH, thyroid stimulating hormone; TPOAb, thyroid peroxidase antibody; TgAb, thyroglobulin antibody; IQR, interquartile range.

### 3.4 Thyroid autoantibodies levels in patients with thyroid dysfunction

Among patients with hyperthyroidism, 9 cases were positive for TgAb and TPOAb, respectively. For patients with hypothyroidism, 3 cases were positive for TgAb and TPOAb, respectively, and among patients with thyroiditis, 6 cases were positive for TgAb and TPOAb, respectively (**Figure 2c**). As shown in **Table 1**, the proportion of thyroid autoantibody positivity in patients with thyroid dysfunction was significantly higher than that in control group, including TgAb (*p*<0.001) and TPOAb (*p*=0.016). The positive rates of TgAb and TPOAb in patients with hyperthyroidism were significantly higher than those in the control group, including TgAb (*p*<0.001) and TPOAb (*p*=0.006). However, there was no statistically significant increase in the proportion of positive TgAb and TPOAb in patients with hypothyroidism compared with those in the control group (*p*>0.05) (**Table 3**). Furthermore, we found that among the four types of thyroid dysfunction, the proportion of positive TgAb in patients with overt hyperthyroidism was significantly higher than that of the other three types (*p*=0.003), while the proportion of positive TPOAb was not significantly different among the four types (**Table 2**).

### 3.5 Risk factors of PD-1 inhibitors-related thyroid dysfunction

**Figure 7:**
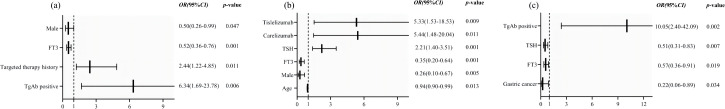
Forest plots of factors associated with PD-1 inhibitor-induced **(a)** thyroid dysfunction, **(b)** hypothyroidism and **(c)** hyperthyroidism.

We compared the baseline characteristics of patients in the PD-1 inhibitor-related thyroid dysfunction group and control group, as shown in **Table 1**. Significant differences were observed in terms of gender (*p*=0.002), chemotherapy history (*p*=0.011), radiotherapy history (*p*=0.015), targeted therapy history (*p*=0.002), type of PD-1 inhibitor (*p*=0.002), primary tumor type (*p*=0.002), baseline FT3 levels (*p*=0.002), and the proportion of positive TgAb (*p*<0.001) and TPOAb (*p*=0.016). However, factors such as age, BMI, baseline FT4 levels, and baseline TSH levels showed no significant differences (*p*>0.05). We included the above statistically significant factors in binary logistic regression analysis, and the results were showed in **Figure 7a**. It was found that male (OR=0.50, 95%CI: 0.26-0.99, *p*=0.047), baseline FT3 levels (OR=0.52, 95%CI: 0.36-0.76, *p*=0.001), positive TgAb (OR=6.34, 95%CI: 1.69-23.78, *p*=0.006) and history of targeted therapy (OR=2.44, 95%CI: 1.22-4.85, *p*=0.011) were associated significantly with PD-1 inhibitor-related thyroid dysfunction.

We further compared the baseline characteristics of patients in the hypothyroidism group and those in control group, as shown in **Table 3**. Significant differences were observed in terms of gender (*p*=0.014), age (*p*=0.02), type of PD-1 inhibitor (*p*=0.008), primary tumor type (*p*<0.001), baseline FT3 levels (*p*=0.003), and baseline TSH levels (*p*<0.001). The statistically significant factors were then included in binary logistic regression analysis. Results showed that male (OR=0.26, 95%CI: 0.10-0.67, *p*=0.005), baseline FT3 levels (OR=0.35, 95%CI: 0.20-0.64, *p*=0.001), age (OR=0.94, 95%CI: 0.90-0.99, *p*=0.013), baseline TSH levels (OR=2.21, 95%CI: 1.40-3.51, *p*=0.001) and type of PD-1 inhibitors (carelizumab, OR=5.44, 95%CI: 1.48-20.04, *p*=0.011, tislelizumab, OR=5.33, 95%CI: 1.53-18.53, *p*=0.009) were associated significantly with PD-1 inhibitor-related hypothyroidism (**Figure 7b**). Additionally, we compared the baseline characteristics of patients in the hyperthyroidism group and those without thyroid dysfunction, as presented in **Table 3**. Significant differences were found between the two groups in gender (*p*=0.011), primary tumor type (*p*=0.007), baseline FT3 levels (*p*=0.042), baseline TSH levels (*p*=0.049), positive rates of TgAb (*p*<0.001), and positive rates of TPOAb (*p*=0.006). By further binary logistic regression analysis, we found that gastric cancer (OR=0.22, 95%CI: 0.06-0.89, *p*=0.034), baseline FT3 levels (OR=0.57, 95%CI: 0.36-0.91, *p*=0.019), baseline TSH levels (OR=0.51, 95%CI: 0.31-0.83, *p*=0.007), positive TgAb (OR=10.05, 95%CI: 2.40-42.09, *p*=0.002) were associated significantly with PD-1 inhibitor-related hyperthyroidism. (**Figure 7c**).

## 4 Discussion

The side effects of ICPIs significantly differ from those of traditional anticancer drugs, involving various organs such as the digestive and respiratory systems, thyroid and pituitary glands, and skin. The endocrine glands, which are richly vascularized and highly sensitive to immune responses, are particularly susceptible during immunotherapy. Thyroid dysfunction is the most common endocrine immune-related adverse reaction associated with ICPIs (2). A 2019 meta-analysis published in JAMA Oncology (8) reported that the incidences of hypothyroidism, hyperthyroidism, and thyroiditis associated with ICPIs were 6.07%, 2.82%, and 0.75%, respectively. Another meta-analysis (9) encompassing 177 clinical trials and 44,595 patients found that the highest incidence of adverse reactions associated with PD-1 inhibitor therapy were hypothyroidism and hyperthyroidism, at 13.7% and 7.5%, respectively. Yoo et al. (9) reported that the incidences of hypothyroidism and hyperthyroidism caused by camrelizumab were 17% and 7%, respectively, while those caused by toripalimab were 18% and 12%, and by sintilimab were 14% and 7%. The study by Shen et al. (10) indicated that the incidence of hypothyroidism and hyperthyroidism induced by tislelizumab were 10% and 4%, respectively. Our study found that the overall incidence of thyroid injury among cancer patients treated with PD-1 inhibitors was 2.97%, with hypothyroidism at 1.20%, hyperthyroidism at 1.77%, and thyroiditis at the lowest rate of 0.50%. Among the four PD-1 inhibitors examined, tislelizumab exhibited the highest incidence at 3.48%, followed by camrelizumab at 3.10%, sintilimab at 2.24%, and toripalimab at 1.75%. Notably, the incidence observed in this study were lower than those reported in the literature. This discrepancy may be attributed to the fact that existing data on thyroid dysfunction caused by PD-1 inhibitors primarily originate from clinical trials, which may not accurately represent real-world populations. Additionally, variations in study methodologies, including sample sizes and screening protocols could also contribute to differences in the incidence.

Thyroid injury induced by ICPIs typically occurs within the initial weeks of immunotherapy, with a median onset time ranging from 18 to 123 days (11). In this study, the median time to thyroid injury in patients receiving PD-1 inhibitor therapy was 67 days, with hyperthyroidism occurring at a median of 69 days and hypothyroidism at 64.5 days. It is noteworthy that 77.31% of the patients with thyroid injury occurred within 4 months. Therefore, it is recommended that routine thyroid function monitoring and evaluation be conducted during the initial 2 to 4 months of PD-1 inhibitor therapy, when the risk of immune-related thyroid injury is high. Additionally, we found that the median time to thyroid injury was significantly shorter in patients with baseline positivity for TgAb and TPOAb (27 days and 50 days, respectively) compared to those who were negative for these antibodies (70 days and 69 days, respectively). This suggested a potential correlation between baseline thyroid autoantibodies and the onset time of PD-1 inhibitor-related thyroid dysfunction. However, given the limited sample size of this study, this hypothesis requires further investigation. In our study, a subset of patients required thyroid-related pharmacotherapy (levothyroxine sodium tablets or methimazole). None of the patients had dose modifications or discontinuation of PD-1 inhibitor therapy due to thyroid-related adverse events. Thyroid function returned to normal levels in 43.7% of patients. The clinical guidelines of the Chinese Endocrine Society (12) indicate that approximately half of patients experience irreversible thyroid function damage, necessitating long-term medication. This underscores the importance of regular thyroid function monitoring during PD-1 inhibitor therapy.

Currently, real-world studies on factors for ICPIs-associated thyroid dysfunction remain limited and inconsistent. Studies suggested that gender was a significant factor influencing the onset of immune-related thyroid dysfunction. A study involving 1,246 patients with malignant melanoma (13) found that female patients were an independent risk factor for thyroid dysfunction. Research by von Itzstein et al. (6) also indicated that female patients were more prone to immune-related thyroid dysfunction. This study found that female gender was an independent risk factor for thyroid dysfunction and hypothyroidism, consistent with the aforementioned results. Mechanistically, several studies (14, 15) suggested that sex hormones influenced immune function, with estrogen potentially enhancing immune cell activity, while progesterone and androgens might suppress immune cell activity. However, Baek et al. (5) through a retrospective analysis of patients treated with ICPIs at Seoul St. Mary’s Hospital, found no significant difference in gender between the normal thyroid function group and the thyroid dysfunction group. Whether female gender is a susceptibility risk factor remains controversial. Additionally, there are differing views on whether age is associated with the occurrence of thyroid adverse events following ICPIs treatment. Study by Campredon et al. (16) suggested that younger patients had a higher incidence of immune-related thyroid dysfunction compared to older patients, while Wu et al. (17) found that age was not a risk factor. In our study, younger age was identified as a risk factor for immune-related hypothyroidism, possibly due to increased susceptibility to immune checkpoint inhibitors in younger patients (18). The largest cohort study on thyroid immune-related adverse events found that elevated baseline TSH levels were associated with the occurrence of clinical hypothyroidism (13). Similarly, Pollack et al. (19) found that high baseline TSH levels were correlated with the risk of thyroid dysfunction, with a baseline TSH level above 2.19 IU/L being a major risk factor for immune-related thyroid dysfunction. Several studies (20, 21) showed that patients with higher baseline TSH levels had a significantly increased risk of developing primary hypothyroidism. The results of this study showed that high baseline TSH was an independent risk factor for immune-related hypothyroidism, consistent with previous literature. Additionally, we found that low baseline TSH levels were significantly associated with the occurrence of hyperthyroidism. These findings indicate that these patients may have subclinical thyroiditis, and PD-1 inhibitors further amplify the inflammatory response, leading to the occurrence of irAEs. However, this hypothesis requires further validation through clinical studies.

Current research (22, 23) showed that the mechanisms of ICPIs-related thyroid dysfunction might involve T-cell-mediated thyrotoxicosis, the tumor microenvironment, and destruction of thyroid tissue by thyroid autoantibodies. Studies (24, 25) have found that after treatment with PD-1 inhibitors, patients exhibited upregulated levels of the pro-inflammatory cytokine IL-2, decreased granulocyte colony-stimulating factor, and elevated granulocyte-macrophage colony-stimulating factor, suggesting that the changes in cytokine levels may be highly correlated with the occurrence of immune-related thyroid injury. The polymorphism of PD-1 gene might be related to the development of irAEs. Orlov et al. (26) found that polymorphism of PD-1 receptor gene might be a contributing factor to thyroiditis following PD-1 inhibitor therapy. Additionally, thyroid autoantibodies are important monitoring indicators for thyroid dysfunction. The study by Osorio et al. (27) suggested a significant correlation between baseline positivity for thyroid autoantibodies and the occurrence of ICPIs-related thyroid dysfunction. Similarly, Okada et al. (28) found that patients with baseline positivity for TgAb or TPOAb had a significantly higher probability of developing thyroid irAEs compared to those who were negative for both antibodies. Brilli et al. (29) also identified baseline positivity for thyroid-related antibodies before ICPIs treatment as an independent risk factor for thyroid dysfunction. In this study, a significant difference in the positivity rate of thyroid-related antibodies was observed between the normal thyroid function group and the thyroid dysfunction group. Baseline TgAb positivity was identified as an independent risk factor for PD-1 inhibitor-related thyroid injury, consistent with the aforementioned studies. This suggests that baseline positivity for thyroid antibodies may be associated with a higher risk of thyroid dysfunction. This may be attributed to the fact that baseline positivity for TgAb or TPOAb indicates inflammation and destruction of thyroid follicular epithelial cells, suggesting a state of autoimmune thyroiditis. When PD-1 inhibitors are administered, they activate systemic T-cell responses, thereby disrupting thyroid immune balance and potentially progressing autoimmune thyroiditis to immune-related thyroid dysfunction. However, the precise relationship between thyroid autoimmune mechanisms and autoantibodies remains unclear, and the underlying mechanisms require further validation. The occurrence of immune-related thyroid injury may also be influenced by the type of tumor. Alqahtani et al. (30) found that cervical cancer patients had a higher incidence of thyroid irAEs compared to patients with lung cancer, breast cancer, or renal cell carcinoma. This study showed that gastric cancer patients had a lower risk of hyperthyroidism compared to patients with lung cancer and other cancer types. Additionally, the results of this study indicated that patients with a history of targeted therapy, low baseline FT3 levels, or those treated with tislelizumab or camrelizumab were more prone to immune-related thyroid dysfunction. This highlights the need for closer monitoring of these high-risk patients when using PD-1 inhibitors in clinical practice.

Some patients experienced transient thyrotoxicosis that later developed into hypothyroidism. In this study, 20 patients (28.2%) initially presented with thyrotoxicosis and gradually progressed to hypothyroidism over the course of the disease, demonstrating a progression consistent with immune-related thyroiditis. The median time to the onset of thyrotoxicosis was 57.5 days, while the median time to the onset of hypothyroidism was 139 days, with a transition period of 68 days from thyrotoxicosis to hypothyroidism. Hypothyroidism and hyperthyroidism induced by PD-1 inhibitors may represent different manifestations of the same pathological process, resulting from destructive thyroiditis or autoimmune destruction of thyroid follicular epithelial cells. This leads to the release of stored thyroid hormones, initially presenting as thyrotoxicosis. When the stored thyroid hormones are depleted and new hormones cannot be produced, hypothyroidism ensues. The Expert Consensus on Immune-Related Adverse Reactions of the Endocrine System Induced by Immune Checkpoint Inhibitors in China (12) states that the transition from thyrotoxicosis to hypothyroidism typically occurs within 2 to 12 weeks. Approximately half of the patients experience irreversible hypothyroidism and require long-term thyroid hormone replacement therapy. Proper management of thyroid injury directly impacts patient prognosis, highlighting the importance of early diagnosis and timely intervention.

This study has several limitations. Firstly, this study is a single-center retrospective case-control design, which may limit the generalizability of the findings. Patient characteristics and treatment protocols often vary across different institutions, and the relatively small sample size from a single center may not fully represent the broader population. To enhance sample diversity and minimize selection bias, future multi-center studies are necessary. Secondly, as current guidelines for the management of immune-related toxicities do not recommend routine testing for thyroid autoantibodies prior to the initiation of ICPIs, further research is needed to determine whether patients with positive thyroid-related antibodies are more prone to developing thyroid dysfunction.

## 5 Conclusions

In conclusion, immune-related thyroid injury is a relatively common yet often overlooked adverse reaction to immunotherapy. In this study, the incidence of PD-1 inhibitor-related thyroid dysfunction was 2.97%, with hypothyroidism at 1.20%, hyperthyroidism at 1.77%, and thyroiditis at 0.50%. Among the evaluated PD-1 inhibitors, tislelizumab demonstrated the highest incidence at 3.48%, followed by camrelizumab at 3.10%, sintilimab at 2.24%, and toripalimab at 1.75%. The median time from immunotherapy initiation to the onset of thyroid dysfunction was 67 days, with median times of 64.5 days for hypothyroidism and 69 days for hyperthyroidism. Notably, 77.31% of cases occurred within the first four months of immunotherapy. Our study found that female gender, lower baseline FT3 levels, a history of targeted therapy, and baseline TgAb positivity were independent risk factors for PD-1 inhibitor-associated thyroid dysfunction. Additionally, higher baseline TSH levels, younger age, and the use of tislelizumab or camrelizumab were associated with a higher risk of immune-related hypothyroidism, whereas lower baseline TSH levels increased the risk of immune-related hyperthyroidism. Given these findings, it is strongly recommended that close clinical and hormonal monitoring be implemented for these high-risk patients prior to and throughout immunotherapy, particularly during the initial 2-4 months of PD-1 inhibitor therapy, when the risk of immune-related thyroid injury is high.

## Data Availability

The original contributions presented in the study are included in the article/supplementary material. Further inquiries can be directed to the corresponding author.
